# Activation of Brain Regions Associated with Working Memory and Inhibitory Control in Patients with Attention-Deficit/Hyperactivity Disorder in Functional Near-Infrared Spectroscopy: A Systematic Review

**DOI:** 10.2174/1573405618666220822101019

**Published:** 2023-05-17

**Authors:** Lihao Hou, Jiaxuan Yang, Lin Xu, Juanjuan Peng, Cho Yin Joyce Law, Tianhao Chen

**Affiliations:** 1 Rehabilitation Department, The Second Affiliated Hospital of Nanjing University of Chinese Medicine, Nanjing, Jiangsu, China;; 2 Pain Department, The Second Affiliated Hospital of Soochow University, Suzhou, Jiangsu, China;; 3 Nanjing Drum Tower Hospital Clinical College of Traditional Chinese and Western Medicine, Nanjing University of Chinese Medicine;; 4 Rehabilitation Department, The People's Hospital of Kaizhou District. Kaizhou, Chongqing, China;; 5 Rehabilitation Department, College of Acupuncture and Massage & College of Health Preservation and Rehabilitation, Nanjing University of Chinese Medicine, Nanjing, Jiangsu, China;; 6 Psychology Department, The Chinese University of Hong Kong, Hong Kong;; 7 Chinese Medicine Department, Hubei College of Chinese Medicine, Jingzhou, Hubei, China

**Keywords:** Functional near-infrared spectroscopy, working memory, inhibitory control, ADHD, systematic review, brain regions

## Abstract

***Introduction*:** Patients with attention-deficit/hyperactivity disorder (ADHD) often show abnormalities related to cognitive activities, especially related to working memory and inhibitory control. Functional near-infrared spectroscopy (fNIRS) is a non-invasive brain imaging technique based on the changes in cerebral hemodynamics to measure the response of brain activities to cognitive tasks.

***Methods*:** In this review, we collected all clinical experiments that evaluated the changes of oxyhemoglobin levels in relevant brain regions of patients with ADHD through cognitive tasks by fNIRS to determine the abnormalities of brain regions related to working memory and inhibitory control activities in patients with ADHD.

***Results*:** From the beginning of November 2021, PubMed, PsycINFO, Scopus, EMBASE, CINAHL, web of science and Cochrane library were searched, and ROBINS-I was a tool to evaluate the quality and risk bias of the articles included. Sixteen eligible clinical trials or randomized controlled trials were included, of which six measured working memory and eleven measured inhibitory control.

***Conclusion*:** We found that compared with healthy people, the activation scope of working memory and inhibition control in the frontal cortex in ADHD patients was smaller than that in healthy people, and the activation degree was weak or even inactive, which can provide new ideas for the direction of research on ADHD.

## 1 INTRODUCTION

Attention-deficit/hyperactivity disorder (ADHD) is a neurodevelopmental disorder that affects 5-7% of preschool-aged children in the world under DSM-IV and often continues into adulthood [1, 2]. ADHD involves a series of persistent problems, such as difficulty maintaining attention, hyperactivity, and impulsive behaviour [2]. In some children, symptoms begin to appear as early as three years old. Due to the patients with ADHD exhibiting persistent and developmentally inappropriate levels of inattention, impulsivity, and hyperactivity, they performed with developing psychological adjustment problems, emotional distress, academic failure, learning disabilities, social interaction difficulties, significant behavioral disturbances, and other developmental disabilities [3, 4].

Cognitive activity is the most common activity in daily human life, which leads to an increase in neuronal activity. In the brain, the increased neuronal activity causes increased energy expenditure [5]. Our common abilities in life include working memory, the ability to inhibit inappropriate reactions or behaviors, and the ability to transfer between different activities. This neuronal activity and blood flow process is called neurovascular coupling [6]. Cognitive activities cause increased brain activation, leading to an increased flow of oxygenated blood (oxygenated haemoglobin, oxy-Hb) and decreased flow of the concentration of deoxygenated blood (deoxygenated haemoglobin, deoxy-Hb) [7]. Moreover, a previous study has revealed that oxy-Hb is a more sensitive indicator of brain activation [8].

Various imaging techniques can measure brain activities by measuring cerebral haemodynamic changes, such as functional magnetic resonance imaging (fMRI), single-photon emission computed tomography (SPECT), and positron emission tomography (PET). Functional near-infrared spectroscopy (fNIRS) has advantages over these imaging techniques because fNIRS is quiet, portable, low-cost, and has a high temporal resolution, especially for children. It can wear on the head to participate in activities in a normal environment instead of lying quietly in a cramped environment [9]. Moreover, fNIRS is compatible with other techniques in a cognitive task, such as magnetic resonance imaging (MRI), electroencephalogram (EEG), and Magnetoencephalography (MEG) [10].

fNIRS is a non-invasive brain imaging technique and a useful tool for measuring brain activities based on the cerebral haemodynamic changes in response to a cognitive task [11]. fNIRS delivers lights of wavelengths in the near-infrared spectrum to the tissue, and the diffusely reflected light is collected and analyzed to assess brain activity based on neurovascular coupling. fNIRS measurement detects changes in the concentration of oxy-Hb and deoxy-Hb in tissue by using illumination at two different wavelengths (780 and 830 nm) [12]. Multi-channel fNIRS is widely used to detect the haemodynamic changes in cognitive activities, which is better for canceling out artifacts among the skin and motion than single-channel [13].

Working memory (WM) is a cognitive capacity responsible for concurrently retaining and processing information [14]. And inhibitory control is a cognitive process in which individuals restrain their impulses and natural, habitual, or dominant behavioral responses to stimuli [15]. Some articles presented that patients with ADHD have weakness in attention control, working memory, and inhibition control [16-19]. Cognitive tasks, which are necessary and essential in our daily life, have different types, including attention, working memory, and inhibitory control tasks, which can be measured by related cognitive tasks [20].

Several tasks measure working memory, such as n-back task, digit span recall tasks, and visuospatial working memory task [21-23]. N-back task is a common test used to measure verbal working memory. In the n-back task, participants need to decide if the current stimulus is the same as the one presented n^th^ steps earlier in a sequence of stimuli. The memory load factor n can be adjusted to make the task more or less challenging [22]. The digit span recall tasks have two versions: the digit span forward test is used to measure the short-term memory and the digit span backward test is used to measure working memory. In the digit span forward test, the participant is asked to recall the exact order of the stimuli being presented, while in the digit span backward test, the participant is required to repeat the presented stimuli in the reverse order as they were presented [21]. A Visuospatial working memory task is used to measure participants' recall of a sequence of visually presented stimuli and the locations of the stimuli in reverse order such as the Corsi block backward recall task [23].

Color Stroop task and Go/Nogo task are the two commonly used tasks to measure the different inhibitory skills [24-26]. Color Stroop task is used to measure the capacity to inhibit cognitive interference. During the task, participants need to read the word that interferes with naming the color (*e.g*., the word ‘GREEN’ appears in red) within the given time [24, 26]. The Go/Nogo task is used to examine participants' inhibitory control. During the task, participants need to respond to Go stimuli (*e.g*., press a button) and withhold the response for nogo stimuli (*e.g*., not press that same button) [25]. The test is passed when the Go condition is met as well as the Nogo condition is ignored.

This is currently the first review to summarize the corresponding brain region activation in ADHD patients measuring working memory and inhibitory control under fNIRS. We aim to provide evidential support to all those engaged in ADHD research.

## 2 METHODS

### 2.1 Literature Search

Electronic databases were systematically searched until November 2021: PubMed, PsycINFO, Scopus, Embase, CINAHL, Web of Science, and Cochrane-Library. The main keywords used were (NIRS OR fNIRS OR near-infrared spectroscopy) AND (ADHD OR Attention deficit OR hyperactivity disorder) AND (working memory OR n-back OR digit span OR block OR inhibitory* OR inhibition* OR Stroop OR Nogo OR go). The review author screened the titles and the abstracts, and the full text of potentially eligible studies was also retrieved and assessed by the Lihao HOU. We have registered our review on the PROSPERO with ID CRD42021289851.

### 2.2 Study Selection

The search keywords were operationalized using a Population, Intervention, Comparison, Outcome (PICO) chart (Table **1**). The population contains all patients with ADHD symptoms. There are no gender, age, and nationality restrictions on the population.

**Fig. (1):**
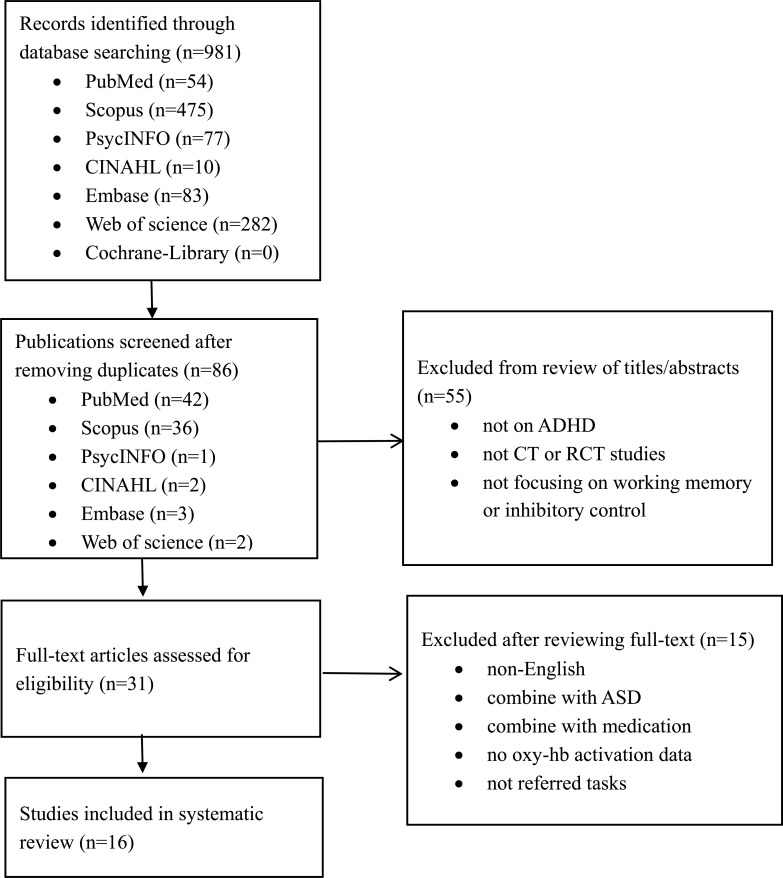
PRISMA flow diagram for data collection and analysis.

Inclusion criteria were defined by PICO: (1) were clinical trials (CTs), including randomized controlled trials (RCTs) and non-RCTs that included ADHD patients; (2) contained the working memory or inhibitory control tasks; (3) used fNIRS to measure for the outcome; (4) were published in English. Studies were excluded if: (1) patients had the medication; (2) combined with the Autistic Spectrum Disorder (ASD); (3) no oxy-hb data; (4) not in English. The process was carried out in line with the guidelines for systematic reviews and meta-analyses (PRISMA) flow diagram (Fig. **1**).

### 2.3 Data Extraction

All data were extracted from studies by the review author according to predefined criteria, which include (1) first author, year, and country; (2) article type (3) number and characteristics of the participants; (3) mean age (4) details of fNIRS devices, (5) region of brain activation and (6) evaluation of scales. For this review, our outcome was the change of oxy-hb level before and after intervention evaluated by fNIRS measures to determine the difference between working memory and inhibitory control activity-related brain region changes in ADHD patients.

### 2.4 Quality Evaluation

The quality of reporting for the trials was evaluated by ROBINS-I (A CochraneRisk Of Bias Assessment Tool: for Non-Randomized Studies of Interventions) [27]. ROBINS-I has seven domains, which cover confounding, selection bias, measurement classification of interventions, deviations from intended interventions, missing data, measurement of outcomes, and results in Fig. (**2**). Each domain was rated as “high” (seriously weakens confidence in the results), “unclear”, or “low” (unlikely to alter the results seriously). Studies that met at least four domains with no severe flaws were considered a low risk of bias. This assessment was processed in Review Manager 5 software (V5.4, The Nordic Cochrane Centre, Copenhagen).

## 3 RESULTS

### 3.1 Studies Inclusion

According to Fig. (**1**), 981 titles and abstracts were retrieved from 7 databases. After adjusting for repetitive and retrospective titles and abstracts, 895 studies were deleted. Then screening for eligibility, 86 abstracts and titles were screened against the inclusion and exclusion criteria. The reasons for the exclusion of 55 studies were as follows: (1) participants not with ADHD (2) not CTs or RCTs (3) not focusing on working memory or inhibitory control. Moreover, 31 full-text articles were further screened, then 15 studies were continued to be excluded for the following reasons: (1) articles are not in English (2) Participants with ADHD combined with ASD (3) Participants with ADHD took medication in the trial (4) fNIRS measurement results had no oxy-hb activation data (5) not referred cognitive activity tasks. Finally, five studies on working memory, ten studies on inhibitory control, and one study on both cognitive activities met the inclusion criteria of this review.

### 3.2 Risk of Bias Evaluation

The risk of bias was assessed and summarized in Fig. (**2**). 11 studies were described as having a low risk of confounding, while five studies had no detailed confounding information [8, 28-31]. Fourteen studies correctly classified intervention status in detail, while two studies cannot exclude the negative impact of medication [29, 32]. Deviation from the intended intervention and comparator groups in 14 studies is regarded as low risk, while two studies are high risk [29, 33]. Six studies that specifically indicated a completion rate of their participants were at low risk of selection bias, while the others gave no details of missing data [30, 31, 34-37]. Moreover, seven studies reported the measurement bias in detail, and others had no information [30, 33, 35, 36, 38-40]. All studies selected the eligible participants and reported results from multiple measurements of the outcome and analyses depending on the findings. Overall, 13 studies met at least four quality domains and were considered low risk of bias, while one study was regarded as having a high risk of bias [29].

### 3.3 Characteristics of the Studies

Table **2** summarizes all the included studies, which focus on the fNIRS measurement of working memory and inhibition control in patients with ADHD. Six of them focused on working memory: three used the visual-spatial working memory task [31, 32, 34], three used the n-back tasks [28, 36, 38], and one used the object working memory task [31]. The other 11 studies focused on inhibition control: seven studies used the go/nogo task [30, 33, 36, 37, 39-41], four studies used the Stroop Color-Word task [8, 35, 41, 42], and one study used the RPS (rock, paper, scissors) task [29]. Moreover, one trial adopted both the go/nogo task and the Stroop Color-Word task [41]. In addition, only one study examined both working memory and inhibitory control [36]. In 16 articles, only one study used the RCT design [38], and the rest trials used the CT design. All of the studies compared ADHD patients with healthy controls, and the sample size ranged from 24 to 86, with a total of 655 participants, and the age ranged from 6 to 47. Most studies used DSM-IV or - V and WISC scales, one article did not mention any scale [42], and the other did not refer to any of the two scales [33]. There are several versions of ADHD rating scale, including ADHD-RS [8, 32, 34], ADHS [31, 33], ADHD index [38], Conners’ RS [32, 35], and WURS [28, 36].

### 3.4 fNIRS Measurement Outcome

In 16 articles, there are many different brands of multi-channel fNIRS devices, such as ETG-100, ETG-4000, OEG-16, NIM, NIRO-300, NIROXCOPE-301, JH-NIRS-BR-05, and the type of multi-channel fNIRS is classified as 4 channels, 16 channels, 24 channels, 32 channels, 44 channels,48 channels, and 52 channels. For multi-channel fNIRS, when there is activation in parts of the brain, oxy-hb increased, and deoxy-hb decreased. Consequently, the two signals are negatively correlated during functional activation, and oxy-hb is often used to reflect the state of brain activation [11, 43]. However, the activation regions of healthy people and ADHD patients are different; patients with ADHD always have lower or no activation levels and areas than healthy people.

According to the working memory, the activation region for verbal working memory focused on the right prefrontal cortex (PFC), especially on the right dorsolateral prefrontal cortex (DLPFC) and ventrolateral prefrontal cortex (VLPFC), while for the visuospatial working memory (VSWM), the activation region is distributed in the right

PFC, frontal pole, left superior frontal cortex (SFS). According to the inhibitory control, the activation region for the capacity to inhibit cognitive interference focused on bilateral PFC, while for the inhibitory control skill, the activation region is widely distributed in bilateral PFC, parietal and frontal regions, left temporal and superior temporal cortex, and right inferior frontal gyrus (IFG) and middle frontal gyrus (MFG).

## 4 DISCUSSION

fNIRS is a portable and potential device for detecting brain activities, which can be widely used in ADHD research. This review aims to summarize the activation of brain regions associated with working memory and inhibitory control in ADHD patients and the differences between ADHD patients and healthy individuals. To our knowledge, no similar systematic review focuses on the association between fNIRS and working memory or inhibitory control in patients with ADHD. A recent systematic review [44] analyzed the application of fNIRS in healthy aging on inhibitory control and working memory. It shows that healthy people experience increased bilateral PFC activation during the n-back and VSWM tasks. While in the Stroop Color-Word task and go/nogo task, healthy people experience increased bilateral PFC activation, especially in VLPFC and DLPFC. The results of the Stroop Color-Word task are consistent, but there are some deviations in other tasks. The n-back tasks represented the activation region on the right PFC, and VSWM tasks also showed the activation area on the right PFC, the frontal pole and left SFS. The go/nogo task showed the increased oxy-hb activation in bilateral PFC and distributed in parietal and frontal regions, left temporal and superior temporal cortex, and right IFG and MFG.

On the one hand, the deviation is related to the number of channels used in fNIRS. The more channels used in fNIRS, the more regions of the brain can be detected [31, 33]. On the other hand, the different wavelengths and correction of artifacts can also affect the measurement results. In addition, the task's workload can also cause differences in the measurement results. For example, Ehlis and colleagues (2007) expressed that the 2-back task showed no right VLPFC activation while occurred in the 1-back task in health control [28]. Two studies indicated that the 2-back task also showed the right DLPFC activation, but the 1-back task showed no oxy-hb region in participants with ADHD [28, 36]. Although participants with ADHD performed worse and showed less or no oxy-hb increase than health control in all tasks, the activation region can also be observed in high workload tasks [28, 36]. However, there was no significant difference in the activation regions among different ages, the activated regions of adults on the same task were essentially in agreement with the activated regions of children.

In 16 articles, there are five articles refer to the method to solve the motion artifacts in oxy-hb data, such as the empirical mode decomposition method [38], a low-pass filter (B0.2 Hz) [32], a moving average method (moving average window: 5 s) [8, 29] and spline interpolation [37]. We try our best to exclude the effect of medication, however, there are still two articles containing a few participants with ADHD taking medicine, such as methylphenidate [29, 32]. Moreover, only one article analyzed the connectivity between brain regions, which found four connection states, two major task-related states, and two task-unrelated states, and distinguished the characteristics of children with TD and ADHD by the occurrence probability of connection states [37]. We believe that this research direction provides a novel way of thinking.

There are several limitations to this review. Due to the language limitations of the researchers, we excluded non-English articles, and these exclusions may result in publication bias. In addition, due to the limited number of such articles, only one RCT was found, and the rest were all CTs with small to medium sample sizes. RCTs provide the highest level of evidence required for confirmation practice, and if there are more randomized controlled trials and large sample experiments, the evidence will be more reliable and effective. Nevertheless, this review has identified brain activation regions for different types of working memory tasks and inhibitory control tasks, which may help explore working memory and inhibitory control mechanisms in patients with ADHD. Future studies could use RCTs with better design quality to provide strong evidence for more ADHD studies in the future.

## CONCLUSION

In healthy people, the activation regions of verbal working memory (VWM) were mainly in the right prefrontal cortex (PFC), especially in the right ventrolateral prefrontal cortex (VLPFC) and dorsolateral prefrontal cortex (DLPFC), while the activation regions of visuospatial working memory (VSWM) were mainly in the right PFC, frontal pole and left upper frontal cortex. Moreover, the activation regions of inhibition of cognitive interference were mainly concentrated in bilateral PFC, while the activation regions of inhibitory control skills were widely distributed in bilateral PFC, parietal, and frontal lobe, left temporal and superior temporal cortex, right inferior frontal gyrus (IFG) and middle frontal gyrus (MFG). While in participants with ADHD, the activation regions are smaller than that of healthy people, and the activation degree is weaker or none in working memory and inhibitory control.

## Figures and Tables

**Fig. (2):**
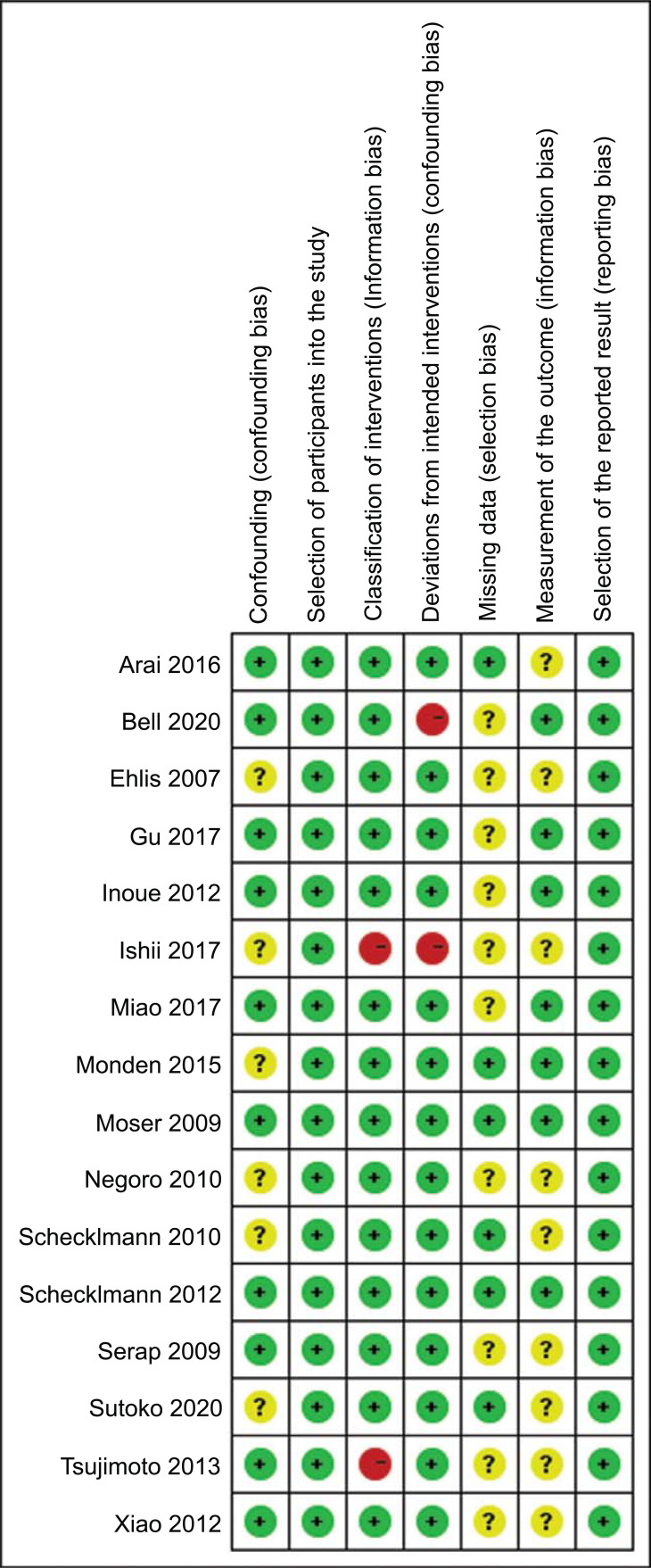
Risk bias for the included studies.

**Table 1:** PICO chart.

**Population**	**Patients with ADHD**
Intervention	fNIRS measurement, working memory and inhibitory control tasks
Comparison	Healthy controls
Outcome	Regions of oxygenate hemoglobin activation

**Table 2:** Characteristics of included studies.

**1st Author, Year**	**Article Type**	**Experimental and Control Type ** **(Sample Size)**	**Mean Age (sd)**	**fNIRS Channels**	**Tasks**	**Region of Brain Activation**	**Scales**
Arai *et al*., 2016	CT	Children with ADHD (n=30) TD children (n=35)	9.23 (1.6)	16 channels (OEG-16; Spectratech Inc., Tokyo, Japan)	SWM task	Experimental: none Control: right LPFC [4ch: p = 0.049; 13ch: p = 0.001], frontal pole [10ch: p = 0.013; 11ch: p = 0.00^8^]	DSM-V; WISC-IV, ADHD- RS
Bell *et al*., 2020	CT	Children with ADHD (n=20) TD children (n=27)	9.84 (1.9)	44 channels (ETG-4000, Hitachi Medical Company, Japan)	go/nogo task	Experimental: right supramarginal and somatosensory cortices [1ch: p = 0.04; 5ch: p = 0.01; 6ch: p = 0.03] Control: bilateral parietal, and left temporal and left STC and bilateral frontal regions [6ch: p = 0.03; 7ch: p = 0.01; 12ch: p = 0.04; 13ch: p = 0.03; 16ch: p = 0.03; 20ch: p = 0.02]	ADHS; CBCL
Ehlis *et al*., 2007	CT	Adults with ADHD (n=13) Healthy adults (n=13)	28.3 (7.2)	24 channels (ETG-100, Hitachi Medical Company, Japan)	1-back task 2-back task	2-back Experimental: right PFC Control: right PFC (higher extent) [8ch,11ch,12ch,22ch: 0.01 < p < 0.05] 1-back Experimental: none Control: right DLPFC and VLPFC [10ch,15ch: p < 0.1; 23ch: p < 0.05]	DSM-IV; WURS-k; HAWIE-R
Gu *et al*., 2017	RCT	Children with ADHD (n=15) TD children (n=16)	7.45 (1.3)	52 channels (ETG-4000, Hitachi Medical Company, Japan)	0-back task 1-back task	1-back: Experimental: right DLPFC and VLPFC Control: right DLPFC and VLPFC (higher extent) [3ch: p = 0.001; 35ch: p = 0.00^6^] 0-back Experimental: none Control: none	DSM-V; WISC-R, ADHD index
Inoue *et al*., 2012	CT	Children with ADHD (n=20) TD children (n=20)	6-14 years	32 channels (NIM Inc., Philadelphia, Pennsylvania, USA)	go/nogo task	go: Experimental: none Control: none Nogo: Experimental: none Control: bilateral PFC [No ch data]	DSM-IV; WISC-IV;
Ishii *et al*., 2017	CT	Children with ADHD (n=18) TD children (n=27)	6-16 years	44 channels (ETG-4000, Hitachi Medical Company, Japan)	RPS tasks	Experimental: none Control: left LPFC and DLPFC [1ch,3ch,5ch,9ch,13ch: p < 0.05]	DSM-IV; WISC;
Miao *et al*., 2017	CT	Children with ADHD (n=14) TD children (n=15)	6-9 years	52 channels (ETG-4000, Hitachi Medical Company, Japan)	go/nogo task	Experimental: none Control: left PFC [37ch: p = 0.038; 48ch: p = 0.008]	DSM-V; WISC;
Monden *et al*., 2015	CT	Children with ADHD (n=30) TD children (n=30)	9.4 (2.45)	44 channels (ETG-4000, Hitachi Medical Company, Japan)	go/nogo task	Experimental: none Control: right IFG and MFG [5ch,6ch,10ch: p < 0.05]	DSM-IV; WISC-III
Moser *et al*., 2009	CT	Children with ADHD (n=12) TD children (n=12)	10.35 (1.75)	4 channels (NIRO-300 spectrometer Hamamatsu Photonics K.K., Japan)	Stroop Color-Word Task	Experimental: PFC, especially in right DLPFC Control: bilateral PFC [No ch data, t = 1.5, P = 0.0^8^]	DSM-IV; Conners’ RS; K-ABC; CPM and SPM
Negoro *et al*., 2010	CT	Children with ADHD (n=20) TD children (n=20)	9.45 (2.02)	48 channels (Hitachi ETG-100, Hitachi Medical Corporation, Tokyo, Japan)	Stroop Color-Word Task	Experimental: none Control: inferior PFC, especially in the inferior LPFC [8ch, 19ch: p < 0.1; 18ch,21ch,22ch: p < 0.00^1^]	DSM-IV; WISC-III; ADHD- RS
Schecklmann *et al*., 2010	CT	Children with ADHD (n=19) TD children (n=19)	11.59 (1.4)	52 channels (ETG-4000, Hitachi Medical Company, Japan)	VSWM task OWM task CON	VSWM and OWM Experimental: left SFS; right DLPFC and VLPFC Control: left SFS; right DLPFC and VLPFC (higher extent) [No ch data, left SFS: t = 2.204; df = 37; p = 0.034; right DLPFC: t = 5.049; df = 37; p < 0.001); right VLPFC: t = 4.185; df = 37; p < 0.00^1^] CON Experimental: none Control: none	DSM-IV; ADHS-DC
Schecklmann *et al*., 2012	CT	Adults with ADHD (n=45) Healthy adults (n=41)	36.26 (10)	52 channels (ETG-4000, Hitachi Medical Company, Japan)	1-back task 2-back task go/nogo task	2-back: Experimental: DLPFC Control: DLPFC (higher extent) [13ch,14ch,18ch,19ch,24ch,25ch,26ch,28ch,29ch,35ch,36ch,39ch,46ch: p < 0.05] 1-back: Experimental: DLPFC Control: DLPFC (higher extent) [15ch,25ch,26ch,35ch,46ch: p < 0.05] go Experimental: none Control: none Nogo Experimental: IFC Control: IFC (higher extent) [3ch,13ch,14ch,24ch,35ch,36ch,38ch,39ch,45ch,46ch,47ch,48ch,49ch,50ch: p < 0.05]	DSM-IV; WURS-k
Serap *et al*., 2009	CT	Adults with ADHD (n=12) Healthy adults (n=12)	31.4 (9.35)	32 channels (NIROXCOPE-301, Hemosoft Inc., Ankara, Turkey)	Stroop Color-Word Task	Experimental: none Control: bilateral PFC [bilateral 1-4ch: p = 0.038]	No refer
Sutoko *et al*., 2020	CT	Children with ADHD (n=21) TD children (n=21)	8.15 (1.75)	44 channels (ETG-4000, Hitachi Medical Company, Japan)	go/nogo task	Experimental: right IFG and MFG Control: right IFG and MFG (higher extent) [No ch data]	WISC-III
Tsujimoto *et al*., 2013	CT	Children with ADHD (n=16) TD children (n=10)	10.59 (1.9)	16 channels (OEG-16; Spectratech Inc., Tokyo, Japan)	VSWM task	Experimental: none Control: right PFC [No ch data; t = 2.39, P = 0.03, d = 0.94]	DSM-IV; WISC-III; ADHD- RS; Conners’ RS
Xiao *et al*., 2012	CT	Children with ADHD (n=16) TD children (n=16)	9.72 (1.46)	16 channels (JH-NIRS-BR-05)	go/nogo task Stroop Color-Word Task	Go/Nogo Experimental: none Control: right PFC [No ch data; t = 2.75, P = 0.00^9^] Stroop task Experimental: none Control: none	DSM-IV; SNAP-IV; WISC-R

**Abbreviations:** ADHD-RS: ADHD-Rating Scale; ch: channel; CPRS: Conners’ Parent Rating Scales; CT: clinical trail; DLPFC: dorsolateral prefrontal cortex; DSM: Diagnostic and Statistical Manual of Mental Disorders; FSIQ: full scale intelligence quotient; IFG: inferior frontal gyrus; LPFC: lateral prefrontal cortex; MFG: middle fontal gyrus; OWM: object working memory; PFC: prefrontal cortex; RCT: random control trail; RPS: rock, paper, scissors; SFS: superior frontal cortex; STC: superior temporal cortex; SWM: spatial working memory; VLPFC: ventrolateral prefrontal cortex; VSWM: visuospatial working memory; WISC-IV: Wechsler Intelligence Scale.

## STANDARDS OF REPORTING

PRISMA guidelines and methodology were followed.

## LIST OF ABBREVIATIONS

ADHD: Attention-Deficit/Hyperactivity Disorder
fMRI: Functional Magnetic Resonance Imaging
fNIRS: Functional Near-infrared Spectroscopy
PET: Positron Emission Tomography
SPECT: Single-Photon Emission Computed Tomography

## SUPPLEMENTARY MATERIAL

PRISMA checklist is available as supplementary material on the publisher’s website along with the published article.
